# Science is a team sport: citations are how we recognize members of the team

**DOI:** 10.1093/g3journal/jkae102

**Published:** 2024-07-08

**Authors:** Lauren M McIntyre

**Affiliations:** Department of Molecular Genetics and Microbiology, University of Florida Genetics Institute, PO Box 100266, Gainesville, FL 32610, USA

From science's earliest days, we have sought to understand the world around us and communicate that understanding to others by summarizing our observations and trying to interpret them in a larger context. Both historically and in modern times, we face challenges in acknowledging the large number of people that contribute to discoveries. Then, as now, sharing credit is not incentivized.

In the early days of genetics as a discipline, De Vries (1900a) and Correns (Deutsche Botanische Gesellschaft 1900) provided critical independent experimentation that helped drive interest in the fundamental concepts of inheritance. Yet, if all we had were these experiments, “Mendel's laws” would not have the central importance they now play in modern science. What remains striking about what we now call Mendelian inheritance is the weight of the evidence—the reproducibility of the observations over time and in different organisms with thousands of experiments performed by different people. We need to recognize and give credit to the many contributions needed to build our understanding of the generalizability and the limits of particular observations. We encourage our authors to cite others who have done experiments in similar systems or with similar hypotheses. We believe strongly in the value of the scientific endeavor and in the importance of the collective knowledge we gain through experimentation across systems and under different conditions and environments. In particular, both journals share a “no scoop” policy. Science benefits when we think about ourselves as a team whose goal is not only to promote our own results but also to place these results in context.

Contextualizing our results is a work greatly facilitated by the collective efforts in the creation of databases and knowledgebases. For example, creating shared vocabulary and common language is an enormous and ongoing collective effort. Whenever we discuss the “molecular function” or “biological process” or the “cellular component” of a gene, we are invoking ontology. Whenever we perform an “enrichment test” based on the Gene Ontology, we are leveraging this collective effort. Initially led by Michael Ashburner (Ashburner *et al*. 2000), the ontology remains an ongoing community effort (Gene Ontology Consortium 2023), and instructions about the latest efforts, how to credit them, and the affiliated tools are found on the website (https://geneontology.org/docs/go-citation-policy/). Whenever a scientist uses the concept of the ontology, these papers should be cited—so that proper credit for the work is measurable and recognizable. The ontology is just one example of collective projects that are valued and often invoked with a web link that directs readers to the resource.

A web link is not a citation. Up-to-date citations for species databases like FlyBase (Öztürk-Çolak *et al.* 2024), the Arabidopsis Information Resource (Reiser *et al*. 2024), the Mouse Genome Informatics (Baldarelli *et al*. 2024), Echoinobase (Telmer *et al*. 2024) and WormBase (Sternberg *et al.* 2024) as well as databases that collect knoledge from multiple sources such as The Alliance of Genome Resources (The Alliance Consortium, 2024) need to be included in the bibliography of your publications. Other examples include the Protein database (Berman *et al*. 2000) and the KEGG (Kanehisa *et al*. 2023). It is important to identify the most recent release, as the curators are continually adding new tools and enhanced functionality as they have for Fungi, S. Pombe Maize (e.g. Basenko 2024; Rutherford *et al*. 2024; Cannon *et al*. 2024). These resources must be cited in the bibliography in order to demonstrate the impact these community resources make on each of our individual efforts. These resources often have tools and algorithms that enhance their functionality. For example PANTHER is an gene ontology enrichment analysis tool (Thomas *et al*. 2022). Acknowledgement for such tools using a web link in the publication does not give credit for the work because these callouts are not indexed and counted as citations. Too, methods are also often ignored in citations. How many times is a correction for false discoveries (FDR) invoked without citation (e.g. Benjamini and Hochberg, 1995)? With an FDR correction, the citation is particularly important because, unlike a Bonferroni correction (Dunn 1959, 1961), there is more than one way to control the FDR (reviewed in Verhoeven *et al*. 2005), and methods that sharpen FDR control continue to be developed (e.g. Stephens 2017).

The *GSA Journals*, and many other journals, encourage complete citation without imposing limits on the number of items cited. The recognition of the central role that community resources, computational methods, and the people behind them play in the success of your research does not detract from the discoveries you have made but increases the reproducibility of your work and elevates the people behind the infrastructure that facilitates our shared endeavor. Our measurable support of these resources is critical to their continued viability.

We encourage you to take the little bit of extra time necessary to give credit where credit is due. We ask that you credit, with a citation in the bibliography, all the databases, knowledgebases, methods, software, and web tools that you have used in your paper.
